# Draft Genome Sequence of an Unusual Ectomycorrhizal Fungus, Pseudotulostoma volvatum

**DOI:** 10.1128/mra.00801-21

**Published:** 2022-02-17

**Authors:** Rachel A. Koch, Christopher E. Bach, Stacy Pirro, M. Catherine Aime

**Affiliations:** a Department of Botany and Plant Pathology, Purdue University, West Lafayette, Indiana, USA; b Department of Biodiversity, Iridian Genomes, Bethesda, Maryland, USA; University of California, Riverside

## Abstract

Here, we report the draft genome sequence of Pseudotulostoma volvatum, an unusual ectomycorrhizal fungus in the “mold” order Eurotiales (Ascomycota, Pezizomycotina). The assembled genome is 60.4 Mbp and contains an estimated 5,492 genes. Compared with closely related species, the *P. volvatum* genome is depauperate in secondary metabolite gene clusters.

## ANNOUNCEMENT

Eurotiales is a fungal order dominated by blue mold- and green mold-forming species, of which a hallmark feature is a large repertoire of secondary metabolite gene clusters within their genomes (1, 2). Within this order, Pseudotulostoma volvatum is an ectomycorrhizal species that has thus far only been collected in the Guiana Shield (3, 4). *P. volvatum* is most closely related to hypogeous, truffle-like species of *Elaphomyces* but differs by producing macroscopic stipitate and volvate fruiting bodies, thus representing the most highly differentiated reproductive structures known within Eurotiales (3). The potential for a high content of secondary metabolite gene clusters and the morphological novelty of this species led us to sequence its genome.

Fruiting bodies of *P. volvatum* were collected in a Dicymbe corymbosa monodominant forest in the Upper Potaro River Basin of the Pakaraima Mountains, Guyana, on 10 June 2015 (voucher specimen MCA 5687/PUL F3438/BRG 41296). DNA for genome sequencing was then extracted from the dried fruiting bodies using the DNeasy plant mini kit (Qiagen, Germany). Using a whole-genome shotgun approach, TruSeq paired-end libraries were generated and sequenced on the HiSeq platform, resulting in 410,349,582 paired-end reads with a read length of 150 bp. For quality control (QC) of raw reads, adapters were trimmed and sequencing contaminants were removed using bbduk (BBTools suite v38.06, https://jgi.doe.gov/data-and-tools/bbtools/). Following QC, reads were normalized with bbnorm to a maximum depth of 60-fold and a minimum depth of 10-fold (BBTools suite v38.06). The resulting normalized reads were *de novo* assembled with SPAdes v3.14 (5) using iterative kmer lengths from 21 to 121 in increments of 10 and run with careful mode. RepeatScout v1.0.6 (6) was used to identify a custom putative repeat library (threshold, >150 occurrences) which was then queried for genomic masking using RepeatMasker v4.0 (7). RepeatMasker revealed that ∼7.7 Mbp was masked, representing 12.74% of the genome. The resulting 60,373,371-bp masked draft assembly comprises 3,416 scaffolds greater than 1,000 bp and has an *N*_50_ value of 45,050 bp and an *L*_50_ of 405 scaffolds. The G+C content of the assembled genome is 45.83%. Using sourmash (8), we were bioinformatically able to determine that no bacterial contaminants were integrated into the *P. volvatum* assembly. The *P. volvatum* genome was then annotated following the MAKER v3.01.03 (9) pipeline. MAKER utilized AUGUSTUS v3.3.2 (10) for *ab initio* gene prediction using the gene training model generated previously for a closely related species, Aspergillus nidulans (GenBank assembly accession GCA_000011425.1) (11), which is also in Eurotiales. We also employed the RNA evidence option in MAKER by providing the associated transcript sequences from Aspergillus nidulans to improve gene prediction quality. MAKER predicted 5,492 protein-coding genes. Functional annotation was accomplished by querying MAKER proteins against UniProt Swiss-Prot database (release 2021_03) using BLASTP v2.9.0 (12). Following gene prediction, genome completeness was assessed with benchmarking universal single-copy orthologs (BUSCOs) v4.1.4 (13) using protein mode and querying the ascomycota_odb10 database. BUSCO determined that this genome contains 89.4% (1,526/1,707) of the BUSCOs, indicating a relatively complete genome.

Twelve putative secondary metabolite gene clusters were identified using antiSMASH 5.0 (14), including three nonribosomal peptide synthetase (NRPSs) or NRPS-like genes, four terpene synthase genes, two polyketide synthase (PKS) or PKS-like genes, and two hybrid NRPS-PKSs. This content is slightly less than the number found in Elaphomyces granulatus (15), but is vastly less than what has been detected in other Eurotiales species, which range from 39 to 81 (1, 2). This draft assembly will facilitate our understanding of the evolution of secondary metabolism and complex fruiting structures within the Eurotiales.

### Data availability.

This whole-genome shotgun project has been deposited at DDBJ/ENA/GenBank under the accession JAHQZX000000000. The version described in this paper is the first version, JAHQZX000000000.1. Raw data are available through the Sequence Read Archive under the accession SRX1936404.

## References

[1] Inglis DO, Binkley J, Skrzypek MS, Arnaud MB, Cerqueira GC, Shah P, Wymore F, Wortman JR, Sherlock G. 2013. Comprehensive annotation of secondary metabolite biosynthetic genes and gene clusters of *Aspergillus nidulans*, *A. fumigatus*, *A. niger* and *A. oryzae*. BMC Microbiol 13:91. doi:10.1186/1471-2180-13-91. 23617571 PMC3689640

[2] Nielsen JC, Grijseels S, Prigent S, Ji B, Dainat J, Nielsen KF, Frisvad JC, Workman M, Nielsen J. 2017. Global analysis of biosynthetic gene clusters reveals vast potential of secondary metabolite production in *Penicillium* species. Nat Microbiol 2:17044. doi:10.1038/nmicrobiol.2017.44. 28368369

[3] Miller OK, Jr., Henkel TW, James TY, Miller SL. 2001. *Pseudotulostoma*, a remarkable new volvate genus in the Elaphomycetaceae from Guyana. Mycol Res 105:1268–1272. doi:10.1017/S095375620100466X.

[4] Henkel TW, James TY, Miller SL, Aime MC, Miller OK, Jr. 2006. The mycorrhizal status of *Pseudotulostoma volvata* (Elaphomycetaceae, Eurotiales, Ascomycota). Mycorrhiza 16:241–244. doi:10.1007/s00572-006-0040-2. 16547736

[5] Bankevich A, Nurk S, Antipov D, Gurevich AA, Dvorkin M, Kulikov AS, Lesin VM, Nikolenko SI, Pham S, Prjibelski AD, Pyshkin AV, Sirotkin AV, Vyahhi N, Tesler G, Alekseyev MA, Pevzner PA. 2012. SPAdes: a new genome assembly algorithm and its applications to single-cell sequencing. J Comput Biol 19:455–477. doi:10.1089/cmb.2012.0021. 22506599 PMC3342519

[6] Price AL, Jones NC, Pevzner PA. 2005. *De novo* identification of repeat families in large genomes. Bioinformatics 21:i351–i358. doi:10.1093/bioinformatics/bti1018. 15961478

[7] Smit AFA, Hubley R, Green P. 2019. RepeatMasker Open-4.0. http://www.repeatmasker.org.

[8] Pierce NT, Irber L, Reiter T, Brooks P, Brown CT. 2019. Large-scale sequence comparisons with *sourmash*. F1000Res 8:1006. doi:10.12688/f1000research.19675.1. 31508216 PMC6720031

[9] Cantarel BL, Korf I, Robb SMC, Parra G, Ross E, Moore B, Holt C, Sánchez Alvarado A, Yandell M. 2008. MAKER: an easy-to-use annotation pipeline designed for emerging model organism genomes. Genome Res 18:188–196. doi:10.1101/gr.6743907. 18025269 PMC2134774

[10] Stanke M, Waack S. 2003. Gene prediction with a hidden Markov model and new intron submodel. Bioinformatics 19:ii215–ii225. doi:10.1093/bioinformatics/btg1080. 14534192

[11] Galagan JE, Calvo SE, Cuomo C, Ma L-J, Wortman JR, Batzoglou S, Lee S-I, Baştürkmen M, Spevak CC, Clutterbuck J, Kapitonov V, Jurka J, Scazzocchio C, Farman M, Butler J, Purcell S, Harris S, Braus GH, Draht O, Busch S, D'Enfert C, Bouchier C, Goldman GH, Bell-Pedersen D, Griffiths-Jones S, Doonan JH, Yu J, Vienken K, Pain A, Freitag M, Selker EU, Archer DB, Peñalva MA, Oakley BR, Momany M, Tanaka T, Kumagai T, Asai K, Machida M, Nierman WC, Denning DW, Caddick M, Hynes M, Paoletti M, Fischer R, Miller B, Dyer P, Sachs MS, Osmani SA, Birren BW. 2005. Sequencing of *Aspergillus nidulans* and comparative analysis with *A. fumigatus* and *A. oryzae*. Nature 438:1105–1115. doi:10.1038/nature04341. 16372000

[12] Altschul SF, Gish W, Miller W, Myers EW, Lipman DJ. 1990. Basic local alignment search tool. J Mol Biol 215:403–410. doi:10.1016/S0022-2836(05)80360-2. 2231712

[13] Simão FA, Waterhouse RM, Ioannidis P, Kriventseva EV, Zdobnov EM. 2015. BUSCO: assessing genome assembly and annotation completeness with single-copy orthologs. Bioinformatics 31:3210–3212. doi:10.1093/bioinformatics/btv351. 26059717

[14] Blin S, Shaw S, Steinke K, Villebro R, Ziemert N, Yup Lee S, Medema MH, Weber T. 2019. antiSMASH 5.0: updates to the secondary metabolite genome mining pipeline. Nucleic Acids Res 47:W81–W87. doi:10.1093/nar/gkz310. 31032519 PMC6602434

[15] Quandt CA, Kohler A, Hesse CN, Sharpton TJ, Martin F, Spatafora JW. 2015. Metagenome sequence of *Elaphomyces granulatus* from sporocarp tissue reveals Ascomycota ectomycorrhizal fingerprints of genome expansion and a Proteobacteria-rich microbiome. Environ Microbiol 17:2952–2968. doi:10.1111/1462-2920.12840. 25753751

